# Dr. Stoker on the Humoral Pathology, &c.

**Published:** 1824-06-01

**Authors:** 


*Pathological Observations, Part* I. *on Dropsy, Purpura, and the Influenza of the latter end of the Year* 1822, *and beginning of that of* 1823; *and particularly on the Morbid Changes of the Blood, and their Influence on the Production and Course of these Diseases, illustrated by select Cases and Dissections.* By WILLIAM STOKER, M. D. Senior Physician to the Fever Hospital and House of Recovery, in Cork Street, &c. &c. Octavo, pp. 244. Dublin, March, 1824.

"Officium Medici circa hominem Ægrum est sanare."

A CONSIDERABLE portion of Dr. Stoker's work is dedicated to the useful purpose of recalling the attention of the profession to the humoral pathology, as it is called, which, for a long period, has been too much neglected. Doubtless it had its share of errors, and perhaps more than its share of absurdities; but still we are convinced that it contained many truths which have been lost sight of since solidism prevailed. Cabanis has traced some of the causes which led to the downfal of the humoral pathology, in the following descriptive but correct sketch of the evils resulting from the *abuse* of one of the most brilliant discoveries in physiology.

"The new light (says he) which was thrown upon the animal economy by this important discovery, (Harvey's Discovery of the Circulation of the Blood) served only to redouble the rage of systems. Nothing else was thought of but to cause the blood to circulate more freely, to destroy its viscosity, to draw off from the body that which was supposed to be corrupted, to purify it, correct it, and renew it, and to preserve the blood-vessels in a relaxed and pervious state. Hence those torrents of aqueous and diluent drinks, with which Bontekoe and his adherents inundated their patients. Hence that sanguinary fury, which the partisans of Botalli thought themselves entitled to exercise in their treatment of all sorts of diseases;—a fury which, though so often damped, in some measure, by systematic murders, has ceased only for intervals, and still from time to time reappears in the schools. Hence, too, that wretched mania of the transfusion of blood, of which the practice almost always deprived those who had the temerity to subject themselves to so dangerous an operation, of their life, or their reason."* 3.

We entirely agree with Dr. Stoker, that the candid enquirer and observer of the ratio symptomatum during life, and appearances post mortem, will frequently be forced to admit the justness of many of those observations on which the humoral pathology was founded—observations which go far to prove that the rise and course of diseases are by no means limited by those lines of demarcation which have so minutely divided the rival systems.

The neglect of the humoral pathology occasioned, of course, a neglect of the chemical analysis of the blood and fluids derived from it, in health and in disease—thus retarding the physiology of the fluids. The late experiments of Brande, Scudamore, and some others, together with the work now under review, will, we hope, invite more general attention to the subject, and lead to improvement in the knowledge and even the treatment of diseases. It must be evident, indeed, that the chyle is affected by the various kinds of food which we use. Indigo gives it a blue colour, madder and beet a red, several vegetables a green. We are informed by Richerand, that when chyle was collected (as at the Veterinary School in Alfort) and exposed to the air, it separated into two parts, the one forming a kind of gelatinous coagulum very thin, and not unlike the buffy coat of the blood—the other in greater quantity and liquid, rising above the coagulum, on its being detached from the sides of the cup to which it adhered. The coagulated mass is semi-transparent, of a light pink colour, and does not resemble the curd of milk, so that the similarity supposed to obtain between milk and chyle, does not exist. The lymph, which constantly unites with the chyle before the latter enters the sanguiferous system, on being received in a vessel by Mascagni, coagulated in eight or ten minutes—turned sour, and separated into two parts, the one more abundant, serous, with a fibrous coagulum in the middle, which, by contracting, formed into a small cake on the surface of the fluid. Hence Mascagni concluded, con-

* Cabanis, pp. 152. Henderson's translation.

trary to Hewson, that lymph consists, for the greatest part, of serum, and that fibrine constitutes its least part.

That the blood undergoes some changes in the portal circulation is very probable; but what those changes are we do not yet know.

"Experiments," says Dr. Stoker, "which I instituted more than twenty years ago, with a view to ascertain what was the difference between the state of the blood in the vena portæ, and of that as it enters the vena cava ascendens from the liver, though rude and incomplete, yet appeared to me to be at least decisive that in health blood is of lighter colour after than before its passage through the liver; and further, that on immersing blood of these different denominations in separate vessels of water at a high temperature, there was a remarkable portion of solid animal matter elevated to the surface of the water in which the blood of the vena cava was immersed, whilst no such appearance presented itself on the water in the other vessel."* 10.

The foregoing observations on humoral pathology are meant to apply chiefly to dropsy, purpura, and certain other diseases in which the symptoms being most palpable to the senses, their seat may be the more accurately determined. Our author begs to premise, that he places different forms of purpura, dropsy, and other diseases of vascular effusion under distinct heads, as appearing to arise in very opposite states of *vital power*, and in distinct conditions of the *fluids or solids*, or both. Those under the first head are denominated dynamic—those of the second, adynamic.†

But all this time we have passed over our author's preface, in which he informs us, that the object of the present publication is to present the profession with some of those facts collected during an extensive experience of 25 years in hospital and private practice. The "warm opposition" which our author anticipates is, we should hope, only imaginary. There is a greater leaning in men's minds towards the humoral pathology than they are willing to acknowledge, and therefore we anticipate a very candid and patient hearing for our author on the part of the public.

In this preface our author introduces a short detail of two cases, which, with many others, led him to undertake the pre-

---

* "See Transactions of the Association of the King's and Queen's College of Physicians in Ireland. Vol. I, page 163 and 4.

† Dynamic from δυναμις, *force*. Adynamic is, of course, the reverse, or rather privation of strength. We do not see that these terms are better than the older ones of sthenic and asthenic.—*Rev.*

sent work. We do not see the very particular bearing of these cases on the subject of the work, but they are interesting in themselves, and therefore we shall notice them in this place.

*Case* 1. *Nov.* 1820. A young lady, aged 11 years, had, in the preceding April, been bled for hepatic and pulmonary disease, threatening general dropsy, and succeeding a severe attack of scarlatina, the blood at the time being deeply and densely covered with buffy coat. Bleeding and mercurials had then been effectual. On going into the country she apparently recovered, and got more corpulent than previously. On the 19th of November, and without any premonitory symptoms, except slight head-ache, she became apoplectic.

"*First visit.*—At seven o'clock in the evening, I found her apparently moribund; the powers of speech, of swallowing, or any kind of voluntary motion were totally suspended. Complete paralysis of the right side had taken place, and convulsions constantly agitated and distorted the other side. The left side of the face was much distorted, the mouth drawn to that side, and the palpebræ of the eye, and the eye itself, agitated with spasm; the muscles of the other eye, which lay open, and the pupil of which was dilated, seemed perfectly paralysed; pulse scarcely perceptible; had frequent and long intermissions; the skin clammy, and unequally warm; respiration quick and laborious.

"Surgeons Macklin and O'Reilly tried to draw blood by openings, made successively in the median vein of each arm, the jugular veins, and temporal artery, but ineffectually, as none flowed from these openings, though assisted by friction and fomentation. Leeches to the temples were then applied; the head was shaved and blistered; stimulant frictions, warm baths, and enemata, were then employed, but all with little apparent advantage, and I left my patient at midnight, though with an injunction to her attendants to persevere in the means in use, and as a *dernier resort* directed a blister to the right hypochondrium, yet with little ground for hope that she could survive the night.

"Second visit.—*November* 20.—Eight o'clock, A. M. I learned that at two o'clock, the pulse having previously become strong and quick, blood began to flow freely from the temporal artery that had been opened in the evening, and soon afterwards the spasms ceased, the powers of speech and of motion were restored, the enemata were returned, bringing fæces along with them; and when about twelve ounces of blood had been discharged from the temple, the opening was secured by bandage, the patient drank freely of whey and fell asleep, continuing to sleep tranquilly to the time of my visit.

"On wakening her none of the symptoms (such as were detailed in the last visit) remained; but there was a high degree of pyrexia, which continued, notwithstanding venesection, and the use of aperients and sudorifics, for several days; and on the fourth day from that of the apoplectic attack, the eruption of measles appeared very generally over her face, body, and extremities, accompanied with the usual pulmonary affection; also with jaundice, and symptoms of derangement in the liver. *Pref.* xiii.

The measles, in this case, were attended with severe pulmonary symptoms and icteric fever; yet she convalesced and returned to the country, where "her recovery went on favourably for some time afterwards." In the succeeding spring, however, our author learnt, with regret, from her medical attendant in the country, that hepatic disease became developed, which was succeeded by ascites, ending fatally in the ensuing summer.

The most remarkable circumstance, we think, in the foregoing case, is the apparently complete state of apoplexy and paralysis, in which Dr. Stoker found the patient. It is manifest, that there was no extravasation of blood, and that the compression could only proceed from turgidity of vessels. But how shall we explain the paralysis on one side?

*Case* 2. A gentleman, 52 years of age, had laboured for 20 years under inconvenient obesity, commencing after the suppression of a gleety discharge from the urethra, and augmented by full living and sedentary habits. He consulted Dr. Stoker in September 1821, on account of lethargic complaints of a growing tendency, notwithstanding the employment of purgatives, blisters, and an issue in the neck. Dr. S. found the obesity excessive, attended with alarming symptoms. The surface of his head, body, and extremities, was every where leucophlegmatic, and the lower extremities œdematous. His lethargic state, for some months previously, had incapacitated him for business, which state was attended with slight head-ache, vertigo, palpitations, and occasional loss of consciousness. His drowsiness was great; yet his appetite was keen, and he had no paralysis. Bleeding from the arm to 40 ounces, and leeches to the temples were repeated six times in the course of the ensuing six weeks—aided by abstemious diet, purgatives, antimonials, &c. By these means every symptom of distress was removed—his bulk was considerably diminished, and his mental and corporeal activity proportionally restored. He returned to his country seat "free from complaint." In the festivities of the ensuing Christmas, however, he returned to his old habits, which were followed by fits partly epileptic and partly apoplectic, leaving him in the intervals paralytic of the left side. When our author saw him in the commencement of 1822, a paroxysm had just taken place which terminated fatally.

We have said that we did not see the particular bearing of these two cases introduced in the preface, on the subject of humoral pathology. That the fluids were deranged in this last case, as well as the solids, there can be no doubt—for what part of the system can escape under intemperance in food and drink, with indolent and sedentary habits? The *plethora ad*

*molem* appeared to play no inconsiderable part in the production of the symptoms in this case. At the same time there cannot be a doubt that there was a vitiated as well as redundant condition of the fluids.

We now pass on to Dr. Stoker's dynamic (or sthenic) cases of dropsy and purpura.

*Case* 1. A lady, 39 years of age, who had suckled nine healthy children, had been always accustomed to regular exercise, and had enjoyed good health, with the exception of occasional attacks of inflammatory sore throat. An attack of this kind, in December 1813, soon after the birth of her sixth child, was attended with severe oppression and difficulty of breathing, followed by soreness and stiffness in the joints of the lower extremities, which state was rapidly succeeded by œdema and purpura from the hips downwards, the purple spots varying from the 10th to 3d of an inch in diameter, being, in some places, confluent. Leeches, aperients, and blisters were employed during the urgency of this attack, and subsequently her health was restored by generous diet, country air, and tonics. She had no return of the complaint till the winter of 1820, while nursing her ninth child. In this attack (preceded by pains of back and limbs, debility, scanty urine, constipation) the purpura was very deep-coloured over every part of the body, and the spots large, especially on the lower extremities. The accompanying anasarca was very general. She would not wean the child. Bleeding was not used. Under the exhibition of saline and mercurial medicines, succeeded by bark and wine, she recovered slowly from the external affections, but general debility continued a long time.

On the 25th December 1821, (while still suckling the same child, with a very scanty supply) she was again attacked with purpura and dropsical symptoms in a very urgent degree—the œdema and purpura pervading every visible part of the body and limbs. The breathing was much oppressed, pulse 90, cough, much languor, tongue white, urine scanty, thirst urgent, pain of back and limbs severe, considerable pain and soreness to the touch in the *right* hypochondrium, the pain shooting towards the shoulder, and increased by the attempt to lie on the *left* side. Twelve ounces of blood were drawn from the arm, with considerable relief, and no tendency to syncope. The blood became buffed almost as soon as drawn. Aperients—expectorants—chicken broth. 26th. The purpura and anasarca have almost disappeared. The nursing was given over, and in a few days she went into the country, with directions to take a mercurial pill every other night, with some sulphate of magnesia every day, for the hepatic complaint. March 29, 1822. On returning from the country Dr. Stoker found considerable fulness in the right hypochondrium, the liver being perceptibly enlarged, very tender to the touch, with pain shooting to the shoulder, and extending towards the left hypochondrium. The face and eyes were jaundiced. Had, in the country, considerable sanguineous and purulent discharges almost daily with the fæces, or separately. She now complains of internal weakness, frequent palpitations, oppression of spirits. Ten leeches to the hypochondrium—six grains of blue pill at night—sulphate of magnesia in the morning. These produced a copious discharge of bilious fæces mixed with grumous blood, numerous ascarides, and purulent matter. The side less uneasy since the application of the leeches. To be rubbed with camphorated oil, and the mercurial pill to be continued at night. Salts in the morning.

When this plan was pursued for three weeks the mouth became slightly affected—the morbid condition of the liver gave way—but the dysenteric discharges continued, and in the succeeding June threatened ulceration of the intestines. The disease seemed to extend to the bladder and kidneys, as the urine itself held purulent matter in suspension, and was passed with great difficulty. All these symptoms gave way under the occasional use of the blue pill, assisted by a mixture of castor oil, oil of turpentine, mucilage, and peppermint water—also by the balsam copaiba. She has since twice become pregnant—and enjoys good health. The next case was not so fortunate. The details are minute and somewhat tedious; but we shall endeavour to give as condensed and connected a view of the case as in our power.

*Case* 2. 20th March, 1820. Mr. S——, aged 60 years, robust, full habit, sanguine temperament, had for many years been affected with hæmorrhoids, from which a copious monthly discharge took place. In the spring of 1819, after a longer interval than usual, he was attacked with very urgent symptoms of inflammation of the lungs and liver, which were relieved by copious bleeding, purgatives, expectorants, and blisters. From that time till within a week of the present date, he enjoyed good health. On the 13th of March he began to complain of pain in his limbs and back, with a general sense of oppression in every part. On the 19th the pains in his joints became very acute, and dark blotches appeared on his legs, his breathing being easily hurried. Castor oil—Dover's powder at night. 20th. Increase of all the symptoms, with an extensive and ill-coloured swelling of the fauces and palate. The integuments on the nose, forehead, cheeks, and ears, were of a jet black colour; the lips were everted, and so swelled, as was also the tongue, that the posterior part of the mouth and throat could not be distinctly seen; "but such parts as were visible, were of a dusky pearl colour, with the cuticle covering them elevated into one great vesicle, and distended with ill-coloured serum."

"Large black maculæ, some of them an inch in diameter, occupied portions of the surface on the arms, especially about the elbows, over the loins and sacrum, and still more extensively over the feet and legs. Both speech and swallowing were almost totally impeded; breathing laborious; pulse oppressed and intermitting; urine scanty, dark coloured and turbid.

"Bleeding being firmly opposed by the friends of the patient, I did not deem it prudent to take the responsibility which, under such unfavorable circumstances, should attach to the employment of that or any other remedy, more especially as the external appearances were such as I had never seen before, and led me to apprehend that gangrene of the parts had already, or would soon commence."* 19.

Dr. Stoker desired a solution of tartar emetic to be given through a tube, till vomiting was excited—an enema of castor oil, turpentine, and barm to be thrown up—a blister to the external fauces—fomentations, gargles, &c. Camphor, jalap and opium to be exhibited through a tube. 21st. The swelling much increased, as is also the dark colour of the purpura—breathing very difficult—speech and swallowing almost entirely annihilated—skin hot and dry—pulse full and laborious in the right arm, feeble and intermitting in the left—thirst urgent.

"The right arm, being tied preparatory to bleeding, swelled suddenly and became livid, so that it was necessary to loose the bandage for a short time. The blood which flowed from the orifice made afterwards in the arm, was remarkably dense and black, becoming buffed on the surface almost as soon as deposited in the cup; but as it flowed the patient breathed, spoke, and swallowed with more and more ease. When about 12 ounces were taken, syncope supervened, the pulse ceasing altogether; but on taking off the bandage, laying the patient in a horizontal posture, and fomenting the epigastrium with flannels and hot spirits, the pulse was soon found to return in the right arm, but not for several hours afterwards in the left; the patient however expressed that he was much relieved." 21.

---

* "The view which I took of the case at this time being influenced by the generally received opinions respecting the nature of Purpura, possibly prevented me from urging the necessity of bleeding, as I otherwise would; the case therefore appears to me the more instructive, and on that account I am led to publish its course with a degree of minuteness of detail which might be deemed unnecessary in ordinary cases." 19.

A blister inter scapulas—sol. sulph. mag. assisted by turpentine enemata—weak Madeira negus. The bandage accidentally came off, and he lost a considerable quantity of blood. Pulse feeble in the right arm—imperceptible in the left. Yet there was no apparent diminution of muscular strength, as the patient was able to get out of bed without difficulty. Purpura unchanged—two bilious stools—urine scanty and turbid—upper extremities anasarcous.

"Half past three o'clock, P. M. Having asked in the morning for a consultation, Doctor John Crampton now met me. The appearance of the face, trunk, and extremities, was now truly formidable, resembling that of a putrifying cadaver inhumed in hot weather; the patient however, could speak and swallow better; the pulse still however continued weak, especially in the left arm. Still however there was no apparent diminution of muscular strength; the purpura on the hips and nates were very extensive, and tangibly elevated at the margin; the urine was scanty, holding extravasated blood in solution; stools were also scanty. The blood taken in the morning appears like one mass of size.

"In addition to the means previously in use, frequent and small doses of a solution of tartarized antimony and crystals of tartar were now prescribed." 22.

In the evening the patient found himself better—pulse firm and 100, but still unequal in the two arms. Three grains of blue pill and one of calomel to be taken every six hours till the bowels are freed. 22d. Rested well in the night—has had four bilious motions—cough and expectoration freer—swallowing and speech improved—purpura the same—swelling about the mouth and fauces reduced—tongue loaded and brown in the centre—upper extremities œdematous. The antimonial solution to be repeated. *Evening*. Has been very ill all day—the breathing most difficult—œdema of the arms increased—yet deglutition is more easy, and the pulse is fuller. Fifteen leeches around the anus—three grains of blue pill, and one of James's powder every four hours. 23d. He died this day, when they were preparing to bleed him. The dyspnœa had increased almost to suffocation, yet the pulse was full and firm. He expired in a sudden convulsion.

*Dissection, five hours after death.* Externally the purple spots presented the same circumscribed extent, and the same jetty hue as before death. The liver was greatly enlarged, completely resembling the "*foie endurcie et engorgée par de matier muqueuse*," of Portal. Two gall-stones in the gall-bladder, each the size of a hazel nut—bile of an ochre colour, and extremely glutinous. Stomach perfectly sound. Large patches of purpura, resembling those on the external surface, were seen on several parts of the mucous membrane of the intestines. No change in the spleen, pancreas, or urinary bladder. In the thorax, the cartilages of the ribs were found firmly ossified. General adhesion of the costal and pulmonary pleura on the right side—slight on the left. No preternatural effusion in the chest or pericardium. Heart of natural size and colour, but loaded with fat. In its right ventricle a firm buffy coagulum was found, three inches in length, one inch and a half in breadth, and half an inch in depth, having two branches extending an inch and a half into the pulmonary arteries. This coagulum was so firm as to require considerable force of the fingers to break it. In every other vessel which was cut into, the blood was found perfectly fluid. No serum or red blood was found in the right ventricle or pulmonary veins. No farther dissection was permitted, and the head was not examined.

### *Reflections by the Author on the above Cases.*

The two cases above-detailed of complicated dropsy and purpura, although they presented some variety in symptoms, predisposing causes, and result, were yet, Dr. S. thinks, sufficiently analogous to be classed together. The *exciting* causes, he believes, were the same in both—a morbid congestion or collection in the mesenteric, hepatic, and hæmorrhoidal vessels, the periodical discharges of which had frequently given relief in the last case. With its total suppression the symptoms of dropsy and purpura commenced. In both cases, oppression and pain preceded the dropsy and purpura—and these indications of partial or general plethora were relieved, though not in the same degree, by bleeding. An affection of the throat was common to both cases. The instances recorded by Sydenham, Darwin, Parry, and Blackall, appear to our author to be of the same nature, viz. *dynamic* dropsy or purpura, to be distinguished from diseases resembling them, and from their own *adynamic* kind, by the following diagnostic symptoms:—

"From Cynanche Maligna, by the absence of ulceration and fœtor in the throat, by not being attended with the same prostration of strength, and by the dropsical effusion being generally synchronous with the cuticular eruption; and lastly, by that siziness of the blood so remarkable in this form of these diseases. Besides that the gums are not spongy, as in scurvy, these forms of disease may be further distinguished by the difference of the exciting causes; one set of symptoms mostly arising from suppressed discharges or eruptions, and the other generally from the privation of fresh vegetable diet or good air.

"The complicated cases of Dynamic Purpura with Dropsy may be distinguished from the Adynamic, by the former being preceded by greater degrees of muscular pains, and by symptoms of plethora, such as general oppression and labouring pulse; and finally, by the buffiness of the blood. The diagnostics of the simple forms of Purpura and

Dropsy will be mentioned subsequently, when these forms of disease are brought under consideration." 31.

The prognosis, in cases of the above kind, may be pretty accurately drawn, our author thinks, from the patient's report of the effects of venesection. If he express relief after the operation, and a corresponding improvement in the state of the pulse takes place, a favourable issue may be expected. On the other hand, if the patient feels much weaker, the pulse becoming more feeble, and the dropsical effusions not abating, the prospect is unfavourable.

That these congestions, or morbid accumulations in the hepatic, mesenteric, and hæmorrhoidal vessels—in other words, in the portal circle; as well as the morbid appearances of the blood itself, arise "from imperfect or irregular sanguification," is considered probable by our author, from a view of the two chief sources of supply to the sanguiferous system, "and the similarity of the fluid (in its colour and properties at these sources, and previously to its being submitted to the action of the lungs or liver) to the buffy coat;" an opinion which he has been further induced to think well founded, from the results of a considerable number of observations lately made, at his request, on blood shortly after it was taken from persons labouring under various forms of disease, and nearly under the same circumstances.

The general oppression and severe articular pains, at the commencement of the cases detailed, together with the dyspnœa and oppressed pulse which succeeded, he attributes to the same cause. But the purpura and œdema, in these cases, seem owing not merely to plethora, but also to a change in the properties of the blood. To this morbid condition of the vital fluid he is also inclined to ascribe the temporary cessation of the pulse, unaccompanied by a corresponding loss of muscular power.

"The *post mortem* examinations which were made in the second case, of the parts previously occupied by disease, discovered no disorganization which could account for the symptoms detailed in the history of the case, and one appearance alone was presented, which could be fairly considered to give a satisfactory explanation of the cause and sudden manner of dissolution; that was the firm buffy coagulum found in the right ventricle of the heart, which was proved in this instance by many striking circumstances to have been formed before death, and also seemed to be a sufficient cause, and therefore to afford a satisfactory explanation of the manner in which that event took place."* 35.

* We have, in another place, expressed our opinion, that the moderns are too sceptical as to the formation of these polypous concretions before death.

The blood drawn in the course of these diseases "*coagulated and became sizy at the same moment, and almost immediately after it flowed into the cups which received it,*" shewing that its sizy surface did not depend on slow coagulation.*

Here Dr. Stoker introduces a tabular view of 27 patients bled for various diseases, in the Fever Hospital; with a minute account of the appearances of the blood, and other circumstances which might be supposed to influence those appearances. These experiments rebut the idea that slow coagulation is necessary to the exhibition of the buffy coat. "The time of coagulation was never proportionate, but often inverse to the extent and degree in which that process took place."

---

We are convinced, from many cases which we have examined, both before and after death, that these polypi do occasionally take place, and produce dreadful effects. Dr. Stoker is evidently of the same opinion.

* A series of experiments has lately been made at the Hotel Dieu, under the eye of Professor Recamier, on the coagulation and buffy coat of the blood, as influenced by the *mode* of drawing it, which deserve particular attention. We shall quote a single experiment as an example.

"2*d of August*. A man, 35 years of age, of athletic constitution, was "selected. After a violent exertion, he was suffering much pain in the lum- "bar region. A vein was opened in each arm, *at the same instant*. In the "*right* arm the orifice in the skin was *one line and a half* (French) in length "—that in the vein *one line*. The stream was continuous, and three inches "in projection, rather weak. The bleeding was stopped at the end of three "minutes. *Left arm*. Orifice in the skin two lines—in the vein one line and "a half—jet very strong, rapid, and continuous, projecting seven inches. "Bleeding stopped at the end of two minutes. *Results*. The blood from "the *right* arm presented no buff. The clot was of the ordinary consis- "tence. The blood from the *left* arm presented a thin layer of buff, the clot and serum being similar to those of the blood from the other vein."

M. Belhomme (the experimenter under M. Recamier) has made about one hundred and fifty experiments on blood drawn in health and in disease. He has come to the conclusion that a medium orifice (one line in the vein)—a strong, rapid, and continuous jet, in the form of an arch—and a narrow vessel for the reception of the blood, are the circumstances most favourable for producing the buffy coat. In strongly inflammatory diseases, however, and in pregnancy, the buffy coat will appear in almost whatever way the blood is drawn. It is a curious fact, first noticed, we believe, by M. Belhomme, that the blood drawn from pregnant women exhales an odour precisely like that which rises from a newly extracted placenta. This, he thinks, may be a criterion of great importance in cases of doubtful pregnancy. We should be afraid to trust to so precarious a diagnostic. The subject, however, deserves attention. We agree with M. Duges in the following remark on the creeds of the Solidists and Humoralists. "Il ne serait pas moins déraisonnable d'adopter des idées toutes contraires, et de nier les alterations du sang, ou de les croire indifferentes aux phénomènes de la santé et de la vie. Un juste milieu entre ces deux extrêmes est, sans doute, la véritable route a suivre."—*Revue Medicale, Mars*, 1824.—Rev.

From these and other facts, our author is naturally led to question the explanation of a buffy coat from the mere subsiding of the red particles during the slow condensation of the lighter parts:—on the contrary, he thinks we are "warranted in supposing an altered and unhealthy state of the blood, exceeding the effects of mere agitation, takes place in the course of the circulation, either from want of due preparation of the fluids at the two chief sources of supply, and of the subsequent changes these fluids should undergo in their passage through the pulmonary, sanguiferous, and hepatic systems, or from the injurious effects of diseased functions in the organs of sanguification"*

"The colour and external characters which designate various kinds of buffy coat, being also found to indicate the particular functions engaged in producing them, afford additional arguments in favour of the foregoing opinions. In simple pneumonia, for example, as appears from inspection of the table, the coat on the blood is generally of a colourless white; but when tinged, it is with bright red, the depth of the tunic, seldom exceeding a few lines, and to this probably it is owing that the cupping on the surface of such blood is generally remarkable; the thin and tenacious film contracting as it forms, and drawing towards the centre the external margin at the circumference of the less contractile crassamentum."

"In simple forms of hepatic disease, on the contrary, the buffy covering is generally darker through its whole substance than in pneumonia, and is externally yellow. It occupies a large proportion of the solid part of the blood, and is not often cupped; when it is cupped, there is reason to suppose that the lungs are partly engaged.

"In diabetic complaints, which there is so much reason to believe originate in imperfect digestion, or insufficient preparation of chyle, it is well known that when blood is drawn it is often found covered over with a whitish milky-like fluid." 40.

In a convessation which our author had with Mr. Todd, Professor of Anatomy and Surgery in the College of Surgeons, he learnt that the opinions which led to the foregoing observations on the diagnostic characters of the buffy coat, coincided with that gentleman's extensive experience. Mr. Todd stated that, in passing through the wards of the hospital, to which he has been for many years surgeon, he could, on inspecting the cups of blood taken in different diseases, be able frequently to pronounce what organs were primarily affected or chiefly engaged in the disease—"that with the white and cupped surface hav-

---

* See Dr. Scudamore's explanation of this circumstance in the review of his work, in the present number.

ing indicated the lungs to be the seat of disease, and that with the dark yellow colour and equal surface the liver."

In making these remarks, our author does not mean to concur in the opinion that the buffy coat is always indicative of inflammation. On the contrary, he has often witnessed much of that appearance on the blood of some kinds of dropsy, where the patient had neither increased action of the vessels, nor any unusual sensation of pain or heat in any part. Again, in certain conditions of the mucous lining of the cavities or of the viscera, when all other characters of inflammation were present, no buffy coat appeared on the blood when drawn.

"I am inclined, from what I have seen in such cases, to think that when inflammation is confined to the mucous membranes, the blood is not generally buffed; and that when it is in a sizy state, then the texture of the liver or lungs is directly engaged, or affected by sympathy with the parts concerned, so as to influence the condition of the functions in these organs." 42.

Our author now relates some cases of dynamic or inflammatory dropsy; but as they are similar to those related by Dr. Crampton and others, we shall only select a single case, as a specimen.

"*April the 7th*, 1823.—Mary Ryan, a mendicant, aged 20, was admitted into the Fever Hospital and House of Recovery in Cork-street, on the 2d inst. labouring under symptoms of congestive Pyrexia, which still continue; skin hot and dry, tongue loaded but soft, complains of headach and restlessness, pain and soreness in the right side; eyes slightly jaundiced. On examination the abdomen is found greatly enlarged, and distended with a fluid, fluctuation being distinctly perceptible; lower extremities anasarcous; has suffered slight indisposition occasionally from amenorrhœa which is of two years standing. Pulse 110 and hard, urine scanty and high coloured.

"Prescribed.—Let 10 ounces of blood be taken from the arm, and let her take five grains of mercurial pill three times a day. Let the abdomen be rubbed with camphorated oil for twenty minutes, three times a day, and swathed with flannel.

"*April the 8th.*—Twelve ounces of blood were taken from the arm in four minutes, and became solid in twelve minutes; it is now covered with a dark yellow and firm buffy coat, slightly cupped, the swelling and tension of the belly, as well as the pain and soreness of the side, are considerably diminished; she also feels much relieved from oppression, and had a good night's rest; urine three pints, depositing a copious brick coloured sediment. Pulse 80, and soft; four bilious stools.

"Prescribed.—Let the pills and friction, as prescribed yesterday, be continued.

"*April the 9th.*—The ascites has been totally removed, neither is there any remnant of infiltration of the lower extremities; rests well, has a good appetite, and is free from complaint; states that the temporary attacks of indisposition with which she has been affected since the amenorrhœa commenced, generally come on monthly.

"Prescribed.—Let her have five grains of compound myrrh pill, and five of sulphate of iron, every second night, and the semicupium twice a week. M. B.

"*April* 26*th*.—Dismissed cured, having enjoyed uninterrupted health since last report. The catamenia, however, had not returned." 62.

This affords a good example of dropsical effusion depending on sanguineous congestion, as well as on an altered condition of the blood—both apparently owing to suppression of the catamenial discharge The immediate relief obtained by bleeding shewed that vascular power was merely suspended, and that when the cause of the distention was removed, the vessels recovered their due calibre and healthy functions.

"Although this is one of the simplest forms, perhaps, of dynamic dropsy arising from congestion, yet it was connected with marks of diseased action in the liver, which also demanded attention; these were the pain and soreness of the right side, the jaundice of the eyes, the loaded and brick coloured sediment in the urine, and the yellow colour of the buffy coat. For these symptoms mercury was employed, as I have deemed that remedy to be always advisable as an assistant to bleeding in dropsies, where the blood is much buffed, but especially where that buff is of a yellow colour." 63.

### Adynamic Dropsy.

Debility, as it regards the loss both of constitutional vigour and vascular power, is the leading and essential characteristic of this kind of dropsy. Yet the line of demarcation between the two will be found frequently broken down, the irruptions being often mutual, so that the morbid symptoms sometimes actually change conditions. Yet, even so, important advantages may arise from having the primitive characteristics of the disease kept in recollection by some prominent designation. Of the various cases related under this head we shall only be able to introduce one or two—and that in a form considerably abridged.

*Case*. *April* 6*th*, 1823.—Margaret Reilly, ætat 32, a mendicant, admitted into hospital on the 24th of March preceding, labouring under dysentery and ascites. The former complaint is relieved—the latter increased. The abdomen is distended and enlarged—sore to the touch—the fluctuation evident in all parts.

"The skin is dry, and of a pale olive colour,* the extremities emaciated, the eyes heavy and slightly jaundiced; pain of right side impedes a full inspiration. The stools are still frequent, but scanty, with severe tenesmus. Pulse 110 and hard, tongue brown; no appetite; rest disturbed; complains much of weakness. The dropsy commenced about three months ago. States that she was delivered of a still-born child, the only one she ever brought forth, almost nine years ago, that the catamenia have not appeared during the last five years, and that she has always suffered severely from poverty, bad diet, cold and nakedness.

"Prescribed.—Let eight ounces of blood be taken from the arm, let her have a draught with castor oil, and twenty drops of tincture of opium immediately; and let her have ten drops of tincture of digitalis three times a day. Let the belly be rubbed with camphorated oil, and swathed with flannel. L. wine four ounces. 55.

She felt immediate relief from the bleeding. The eight ounces were drawn in three minutes, and became solid in fifteen; yet the coagulation was not firm at the end of 24 hours—no buff—the surface bluish, and very serous. The tension and pain of belly, together with the ascites, much abated—skin soft and better coloured—three pints of urine in the last 24 hours, straw-coloured and void of sediment—tongue cleaner—feels an appetite—slept well last night—four stools, with tenesmus. 8th. The ascites still less, but there is acute pain in the region of the heart—pulse 100, and hard. Venesection to eight ounces, and a blister to the right hypochondrium—seven grains of Dover's powder, 6$^{tis.}$ horis. 9th. Nine ounces of blood were taken yesterday with relief. It did not firmly coagulate—the surface was interspersed with small patches of yellow frothy buff—no return of pain since the bleeding—ascites further diminished—three quarts of urine since yesterday—tongue clean—appetite good—four stools with tenesmus. Eight grains of Dover's powder at night—twelve drops of tincture of digitalis twice a day. 10th. Ascites rather increased—skin and eyes slightly jaundiced. Blue pill, opium, and digitalis every eight hours. 12th. Things are amended—tongue clean, pulse natural—two natural stools. Medicines continued. 13th. Dysenteric symptoms returned, but no dropsical symptoms. These were removed by castor oil, opium, and mercury. Her health gradually improved under these medicines for the ensuing six weeks, the mercury being continued till the mouth became affected. She afterwards had a relapse, and required bleeding,

* "This is the nearest description which I can give of that more than sallow colour of the skin, which characterizes the dropsy of debility so often to be met with amongst the poor of Dublin."

mercury, and digitalis, with occasional opiates. She was dismissed cured on the 10th May.

Our author observes that, in this case, the congestion which led to the hydropic effusion was produced by two kinds of causes—1st, those long applied, in debilitating the vascular system—and 2dly, suppression of the various secretions, by which certain portions of the circulating mass are carried out of the system.

"That pale olive colour of the skin which accompanied this case, and which so remarkably characterizes those dropsies of debility, which are to be met with amongst our poor, as well as the jaundice of dropsies, is very generally found connected with diseased liver, and appears to me to arise from want either of due separation, or of complete union of the hydrocarbonous principles of the venous blood, which should take place in its passage through the liver. For the vessels of that organ, naturally tardy in their action, may be well supposed to be readily influenced by the debility which in such cases pervades the vascular system in general.

"This case appears to me instructive not only as indicating the *ratio symptomatum*, but as exhibiting an exquisite example of Adynamic dropsy changing its nature, under circumstances in which, according to very generally received opinions, that might be least expected to happen; for though combined with dysentery in general, a debilitating disease, yet it assumed such a character as to be relieved only by repeated bloodletting." 73.

We cannot follow Dr. Stoker through his train of pathological reflections on this case, though the reader will certainly benefit by their perusal. We shall give, in conclusion, a short abstract of a long case, as there was an instructive dissection at the end.

*Case.* Elizabeth Lake, a mendicant, aged 24 years, had been admitted into the Fever Hospital on the 4th of March, labouring under obstinate dysentery. This, in some measure, gave way, after a variety of remedies had been used—but no mercury. On the 5th of April she came under Dr. Stoker's care. She then complained of general oppression, pain of back, tenesmus, want of rest. The integuments of the face, arms, lower extremities, and abdomen, all distended with water—the fluid gravitating towards the side on which she happened to lie. Ascites was also unequivocal—abdomen painful to the touch. This dropsical effusion had come on as soon as the dysentery was checked. Thirst urgent—tongue white—urine scanty—pulse 100 and hard—palpitation and pain at the heart. She had been long subject to piles. Venesection to eight ounces—an aperient. 6th. Felt almost immediate relief from the bleeding. The blood coagulated in 15 minutes, with a covering of a bluish but not firm texture, interspersed with a few spots of yellow buff. The abdomen is less painful since the bleeding—the anasarca diminished in every part. Three grains of blue pill, one of digitalis, and half a grain of opium to be taken thrice a day—a blister to the right hypochondrium. Dysentery now returned, and this they were unable to check. She died on the 24th of April.

*Dissection.* The cavity of the peritoneum contained about two quarts of a fluid, like whey, intermixed with portions of gelatinous lymph—resembling that found in an extensive chronic abscess. Internal surface of the peritoneum streaked, in many places, with red vessels, or coated with adherent lymph. The folds of the small intestines adhered together. Several hard livid tumours, varying in size from a crown piece to a sixpence, dispersed over the jejunum, which was morbidly thickened. On the internal surface of the intestine these tumours exhibited bloody points, and ulceration of the mucous membrane. Liver, spleen, pancreas, and kidnies healthy. In the thorax the lungs were found healthy—the heart adhering by soft gelatinous lymph to the internal surface of the pericardium at every point—its surface soft, and the organ smaller than usual.

In this case it will be seen that the inflammatory action supervened on dysentery; and, when relieved by bleeding, the dropsical effusion considerably abated. The nature of the abdominal and pericardiac effusion shewed it to be decidedly of an inflammatory kind. The true and original seat of the disease, Dr. Stoker thinks, was in the small intestines—"the hard livid tumours being largest and most numerous near the duodenum, and, unless when ulcerated, being covered with the serous membrane of the intestines."

"Here the incipient disease was discovered to be in livid tumours, exactly resembling piles after death, for so the anatomist expressed, as soon as he saw them; the colour of each tumour, as well as its seat in the vascular coat of the intestines, manifesting the accumulation of the blood on which it depended.

"The obstruction to the flow of blood, which led to its accumulation, may arise from loss of power in the vessels, or from cohesiveness of the blood, or from both these causes together, which last condition I believe to be the most frequent; and that the tumours, in this instance, were connected with loss of vascular power, as well as with morbid cohesiveness of the blood, is, I think, rendered probable by recollecting that the piles which this patient stated she had laboured under many months previously to the dysentery, were preceded by a long protracted fever, and that it was during recovery from it that the hæmorrhoidal excrescences appeared. The analogy between these tumours and piles would also lead us to a supposition, that they were connected partly with siziness of blood, as every practitioner in medicine of any experience must have observed how generally blood is buffed when drawn from those disposed to piles." 107.

We must pass almost entirely over Dr. Stoker's cases and observations on *adynamic and contagious* purpura, as our limits are drawing to a close. The subject of purpura too has been touched upon at the beginning of this article. The following case is an epitome of our author's *adynamic* instances.

*Case.* Nov. 19. Tobias Barry, aged 13, was received into Sir Patrick Dun's hospital, under Dr. Osborne. His skin was sprinkled over with numerous small maculæ of the colour of port wine, accompanied by a constant hæmorrhage from his gums, and a soreness in his throat. Pulse 132 and weak—bowels natural. This hæmorrhage had lasted (more or less) for six or seven days—the maculæ, however, were not observed till the day before admission. A blister—sulphuric acid—tincture of digitalis. 21st. The maculæ of a brighter colour—pulse 124—has had two fainting fits. 22d. Maculæ the same as before—has an offensive odour about him—breathing impeded, with a hoarse dry cough. 23d. The hæmorrhage rather diminished, but he died this day, without any violent symptoms.

*Dissection.* The spots on the skin were found to be produced by blood effused into the substance of the cutis, and not on its surface under the cuticle, as has been supposed.

"Spots, precisely resembling in appearance and mode of formation were found on the pleura, substance of the lungs, the pericardium, the surfaces and interior of the muscular substance of the heart, in the abdominal muscles, diaphragm, and all the muscles that were cut through in the course of the dissection, in the peritoneum, the substance of every part of the alimentary canal, and on its internal surface. Very few spots were found in the liver, kidnies, or spleen; and the latter organ was rather more firm than usual, and slightly enlarged, but its texture and colour perfectly natural. The bladder of urine had suffered most from the disease, the blood having been effused so largely into its substance that the internal tunic was elevated into a number of regular dark coloured folds, which were so prominent as to materially diminish the cavity of the bladder. Some liquid blood was also found in it. With the exception of the above morbid appearances the viscera were perfectly healthy. A very few spots were seen in the integuments of the cranium and dura mater, but none in the substance of the brain. The blood throughout the body was remarkably fluid; and in place of any fulness of either the venous or arterial system there appeared to be rather a deficiency of the circulating fluid." 147.

Our author remarks that, viewed in contrast with dynamic purpura, this case shews how much such forms depend on a dissolved state of the blood, as well as relaxation of the vessels allowing of its transmission through minute exhalents, permeable only by colourless fluids in a healthy state.

### *Treatment of Dropsy and Purpura.*

Most of the principles of treatment have been anticipated in the foregoing pages, while stating the cases. The practical remarks therefore under the present head may be brief. Dr. Stoker has a short section on prophylaxis, in which he shews the great importance of regulating the ingesta and egesta—or in other words, the functions of the chylopoietic organs. The Roman satirist observed the pernicious effects of indulgence in the pleasures of the table*—though there is strong reason to believe he liked his cup of *Falernian* as well as any man now living. In respect to medicinal treatment, Dr. Stoker places blood-letting, local and general, at the head of the remedial agents. Notwithstanding the excellent writings which have appeared from the pens of Blackall and others, there is still great prejudice against, or rather terror of the lancet in cases of dropsy and purpura. There is no doubt, however, among the well-informed practitioners, that two states or diatheses, of a very opposite kind, exist in these diseases—one of vascular power, and the other of debility, requiring very different modes of treatment. In the former, cautious blood-letting is highly beneficial—in the latter it is often, if not generally, injurious.

"The chief points to be ascertained in employing venesection in dropsy and purpura, appear to me to be first, the nature of the existing causes, whether such as would affect the quantity or quality of the circulating mass, either by obstruction to its natural outlets, or injury of any of the functions by which its healthy state was preserved.—Secondly, that the state of muscular power, or capability of vascular action, as evinced by capability of exertion, and by a vigorous resistance in the vessels to the morbid accumulation of their contents be not materially impaired.—And, lastly, that the patient expresses relief from the blood drawn on the preceding indications, that it be firmly coagulated, and if buffed, it will afford additional grounds for hope of further relief by the repetition of the remedy.

"The indications therefore of blood-letting in dropsy and purpura should be, strength of the patient not materially diminished, increased vascular action evinced by strong pulse and increased temperature, relief

---

* "Crescit indulgens sibi dirus hydrops,

"Nec sitim pellet, &c."—Hor.

of oppression, and of pain immediately felt after the operation, and the subsidence afterwards, though not for a few hours, perhaps, of the dropsical swellings." 159.

Our author very properly advises caution in blood-letting, in dropsies where the cavities engaged contain a vital organ. Thus in hydropericardium, there may be such a temporary increase of effusion, during the excitement which succeeds venesection, as to overwhelm the heart and destroy its function. When such a cavity has appeared distended with hydropic fluid, of the dynamic kind, our author has always thought it advisable to precede venesection by topical bleeding and blisters, and by the internal use of digitalis and antimony.

The modus operandi of blood-letting, both local and general, in relieving dropsy, is elucidated, our author thinks, on the principle of increased absorption, as insisted on by Dr. Paris, Magendie, and others.*

The modi operandi of blisters, antimonials, mercurials, digitalis, need little comment. Our author traces an intimate connexion between the diseases under consideration and biliary derangement—hence he strenuously recommends blisters to the right hypochondrium as of extraordinary efficacy "in tendencies to dropsical or purpural effusion."

"The symptom accompanying such effusive tendencies, which has led me chiefly to connect them with diseased function of the liver, and thence to this remedy, demands particular notice on that account, and also as one always indicating extreme danger, and sometimes offering a fatal prognostic.

"This symptom is either obstinate grass green vomiting, such as attended on some of the worst cases both of purpura and dropsy admitted latterly into our hospital, or the still deeper green alvine discharges which so generally accompany hydrocephalus, and effusions on the brain in bad fevers." 167.

We perfectly agree with Dr. Stoker in his remarks on the *chemical theory* which has been set forth respecting those green stools which we so frequently see in the diseases of children.

---

* Our tropical practitioners long ago observed that congestion or plethora about the head prevented the absorption and action of remedies, as of mercury for instance;—and they bled to remove this plethora and accelerate the introduction of remedies. "This oppressed state of the sensorium renders the absorbent system so torpid that there is no chance of mercury being taken into the constitution. *Evacuations, under these circumstances, by relieving the brain, invariably accelerate ptyalism.*" Johnson on Tropical Climates, 1st Edition (1813) page 207. This passage unequivocally proves that the experiments of Magendie and the remarks of Dr. Paris were anticipated by observations made at the bed-side long before.

If they were attributable *merely to acidities* in the primæ viæ we should have them much more frequently. The theory is entirely negatived by the fact that in dyspeptic subjects, who are harrassed with acidities in the primæ viæ we rarely have green—and very often pale or clay-coloured stools. Others have attributed these spinage-looking evacuations to the calomel administered. We have repeatedly seen them where no calomel had been given. We consider them as *disordered secretions* originally, whose colour may possibly be modified by medicines taken into, or acids generated in the digestive organs, but which cannot be produced from healthy secretions by either of the above agencies. If examined, they will be found entirely destitute of the real fœcal smell.

"For without some morbid change, the accidental admixture of an acid would not produce a green colour; such as Doctor Heberden in his commentaries asserts, he found in the urine of a certain jaundiced patient, which was, when first passed, of a deep yellow. I am farther warranted, I think, in this opinion, by the absence of such green colour, unless accompanied by urgent symptoms of disorder in the hepatic system. If this colour was produced simply by mixing an acid with mere bile, that symptom must be as frequent as acidity in the *primæ viæ*, which is well known not to be the case." 175.

*Antimonials.* Dr. Stoker has a high opinion of antimony in dropsical diseases, as stimulating the various secretory organs to a better discharge of their functions. He has combined antimonials with mercury in dropsies, with much advantage. And in the cure of certain stages of dropsy, where tonics were indicated, but where their employment alone excited fever, as cinchona, steel, &c. he found that inconvenience frequently corrected by the addition of antimonials.

*Mercury.* This, of course, is an important and old-established remedy in dropsies. It may, either alone or combined with other medicines, be directed to various organs, and made purgative, diuretic, sudorific, or expectorant.

"It possesses however, other qualities besides those of mere stimulants which extend the scope of its utility in some forms of those diseases, whilst they limit it in others.

"These other qualities which I mean are, that of entering and changing the condition of the mass of blood itself, and of interrupting such a morbid train of actions in the functions of the part affected, as habit, in proportion to the time of its continuance, had tended to establish.

"When the intention is to promote or increase the alvine discharge, mercury, under the form of calomel, may be usefully employed, combined with jalap, scammony, gamboge, or elaterium, according to the degree of the purgative effect which the prescriber proposes, and under the same form its stimulus may be combined with squills or antimonials, so as to increase the sorbefacient, expectorant, sudorific or diuretic effects of these remedies.

"It is however in those cases of effusive diseases, connected with sizy blood arising from imperfect sanguification in the lungs or in the liver, but especially in the latter, that mercury can be beneficially employed in such a manner as to enter the sanguiferous system, and thus influence the condition of the blood itself, as well as that of the function engaged. When administered with a secondary intention, mercury, reduced to an oxyde by trituration, given internally, or by external friction, is to be preferred; and in cases of laxity of the bowels, may be advantageously combined with opium." 179.

With the above extract we must conclude our review of Dr. Stoker's work, which contains more of good practical matter than ingenious reasoning—especially in these days of anti-humoral pathology. In the composition, Dr. S. has been rather inattentive, and errors of the press are to be seen in almost every page. These, of course, we consider as very trifling blemishes, but still, as they are so easily obviated they ought to be avoided. We shall again pay our respects to Dr. Stoker, in our next Number, when reviewing his Observations on the Epidemic Influenza of 1822-3. In the mean time we part from him with feelings of much respect and consideration.

